# Study on the Relationship Between Respiratory Distress Syndrome and *SP-A1* (rs1059057) Gene Polymorphism in Mongolian Very Premature Infants

**DOI:** 10.3389/fped.2020.00081

**Published:** 2020-03-17

**Authors:** Xiaoli Wang, Yuheng Zhang, Hua Mei, Caiyan An, Chunzhi Liu, Yayu Zhang, Yanbo Zhang, Chun Xin

**Affiliations:** ^1^Division of Neonatology, Department of Pediatric, The Affiliated Hospital of Inner Mongolia Medical University, Hohhot, China; ^2^Clinical Medical Research Center of the Affiliated Hospital, Inner Mongolia Medical University, Hohhot, China

**Keywords:** respiratory distress syndrome (RDS), pulmonary surfactant protein A1 (SP-A1), gene polymorphism, Mongolian very premature infants, respiratory tract management

## Abstract

**Aim:** To study the relationship between rs1059057 polymorphism of pulmonary surfactant protein A1 (SP-A1) and respiratory distress syndrome (RDS) in Mongolian very premature infants.

**Methods:** Applying the strategy of case-control study, 120 Mongolian RDS very premature infants (58 males and 62 females) in the western part of Inner Mongolia were selected as the case group, and 120 subjects of non-RDS very premature infants (56 males and 64 females) with the same nationality, same sex and similar gestational age were used as the control group. The single nucleotide polymorphism (SNP) site rs1059057 of *SP-A1* was genotyped using polymerase chain reaction-single strand conformational polymorphism (PCR-SSCP).

**Results:** Two genotypes, *A/G* and *A/A*, were detected at the *SP-A1* rs1059057 locus in the western part of Inner Mongolia. In the case group, the frequencies of two genotypes were 53 and 47%, and the frequencies of *A* allele and *G* allele were 73 and 27%, respectively. In the control group, the frequencies of the two genotypes were 42 and 58%, and the frequencies of *A* allele and *G* allele were 79 and 21%, respectively. There was no significant difference in the genotype frequency of *SP-A1* (rs1059057) locus between the case group and the control group (*X*^2^ = 3.275, *P* > 0.05), and no significant difference in allele frequency between the case group and the control group (*X*^2^ = 2.255, *P* > 0.05).

**Conclusion:** The genotypes and allele frequencies of *SP-A1* (rs1059057) locus were not associated with the incidence of RDS in Mongolian very premature infants in western Inner Mongolia.

## Introduction

Neonatal respiratory distress syndrome (NRDS), also known as hyaline membrane disease, is mainly due to the lack of pulmonary surfactant (PS), which leads to an increase in alveolar wall surface tension and decreased pulmonary compliance, and initiates newborn's sexual dyspnea shortly after birth, even clinical syndromes of respiratory failure (1). NRDS often occurs in premature infants, especially in premature infants within 34 weeks (2). The probability of NRDS in premature infants with gestational age within 28 weeks is up to 80% (3, 4). At present, international and domestic medical technologies are developing rapidly, and the levels of medical treatment in neonatal ward are also constantly improving. The survival problem of NRDS children has been basically solved, yet prognosis and treatment have remained difficult. Therefore, the respiratory tract management problems of children with NRDS should not be underestimated, we still need to continue to strive our efforts on them.

Pulmonary surfactant protein-A1 is a kind of alveolar cell surfactant protein synthesized and released by type II alveolar epithelium. The causes of NRDS are very complicated and there are many different opinions nowdays, but many studies have found that the lack and abnormal changes of SP-A1 are the main cause of NRDS (5–7). Studies have demonstrated that the incidence of NRDS may be related to *SP-A* gene polymorphism (8, 9). Through the cDNA sequence analysis of SP-A1, it is confirmed that SP-A1 has four alleles (6A, 6A^2^, 6A^3^, and 6A^4^), and different gene mutations can make the expression of SP-A1 abnormal, which leads to the occurrence of respiratory diseases (10). It is worth noting that there are some differences in the relationship between SP-A gene polymorphism and diseases among different regions, races, and ethnic groups (11). Therefore, our team detected the polymorphism of *SP-A1* gene locus in Mongolian very premature infants in western Inner Mongolia to explore its role in NRDS etiology. This paper mainly introduces the relationship between the gene polymorphism rs1059057 of *SP-A1* and the RDS of Mongolian very premature infants, in order to provide help for the rescue of Mongolian very premature infants in the western part of Inner Mongolia.

## Subjects and Methods

### Subjects

Applying the strategy of case-control study, one hundred and twenty Mongolian RDS very premature infants (58 males and 62 females) who were hospitalized in the department of neonatal pediatrics in our hospital from January 2012 to January 2019 were selected as the case group. The selection criteria were as follows: ➀ the immediate family members have lived in Mongolians in the western part of Inner Mongolia for at least three generations. ➁ The sex ratio was roughly balanced, the birth weight was 0.138~ 0.184 kg, and the birth weight was 28^+3^ weeks ≤ the gestational age <32 weeks. ➂ In accordance with the diagnostic criteria of RDS issued in Europe (4). One hundred and twenty Mongolian non-RDS very early infants (56 males and 64 females) who were hospitalized in the department of neonatal pediatrics in our hospital with the same period, race, sex, and gestational age were selected as the control group. The subjects were selected according to the following criteria: ➀ the immediate family members have lived in Mongolians in the western part of Inner Mongolia for at least three generations. ➁ The sex ratio was roughly balanced, the birth weight was 0.142~0.190 kg, 29^+1^ week ≤ the gestational age <32 weeks. ➂ The common chest X-ray showed no pulmonary inflammation and RDS, and the blood routine and C-reactive protein examination showed no obvious infection.

The following subjects were excluded: ➀ congenital or genetic metabolic diseases; ➁ laboratory examination showed severe infection; ➂ severe history of intrauterine or postnatal asphyxia; ➃ gestational diabetes mellitus; ➄ other diseases that may be accompanied by respiratory symptoms; ➅ there are other diseases and related factors that may affect the experimental results.

### Materials and Reagents

Blood Genome DNA extraction Kit and the main regents of PCR were from Sangon Bioengineering Co., Ltd (Shanghai, China). 6 × DNA Loading Dye and DNA Ladder Mix (100–10,000 bp) were from ThemoFisher (R0611 and SM0332). SanPrep column PCR product purification kit was also from Sangon Bioengineering Co., Ltd (Shanghai, China). Main reagent of DNA sequencing were from ThermoFisher (Applied Biosystems™).

### Sample Collection and Processing

The venous blood of Mongolian RDS very premature infants and non-RDS very premature infants in western Inner Mongolia was collected and stored at −80°C. At the same time, the clinical data of gestational age, sex, and birth weight of very premature infants in the experimental group were collected.

### Extraction and Detection of Genome DNA From Samples

The sample DNA was extracted strictly according to the instructions of genomic DNA extraction kit (Sangon Bioengineering Co., Ltd, Shanghai) and the extracted DNA was stored at −20°C. DNA quality detection: ➀ Five microliter of DNA solution was loaded to a 1% agarose gel and the gel was run in 1xTAE at 120 V. A single clear band indicates the extracted DNA to be intact and of sufficient concentration for a PCR reaction. ➁ The concentration and purity were detected by spectrophotometer, and 1 μL DNA solution was loaded to Nanodrop to determine the OD values. A value of OD260/280 between 1.7 and 2.0 demonstrates a reliable quality of the extracted DNA.

### *SP-A1* Gene Polymorphism

We selected rs1059057 of SP-A1 as our study SNP, according to the following reasons: ➀ SNP database Genbank (http://www.Ncbi.Gov/genBank) provides a number of SNP research sites located in the first functional gene (*SP-A1*) of *SP-A*, in which the site studied in this paper is numbered as rs1059057 in the genebank dbSNP database. ➁ Global minor allele frequency (GMAF) of rs1059057 is 0.08(>5%), and the minimum allele >5% meets the basic conditions for SNP selecting. ➂ Located in exon 6 of this gene coding region.

### PCR Amplification

➀ Template: zero point five to one microliter of blood containing anticoagulant (EDTAK2) was directly added to 20 μL PCR reaction system. ➁ Paraffin-embedded tissue samples: a single 10 μm paraffin section was treated with 50–200 μL PCR reaction buffer containing 0.2 mg/ml protease K. The volume of buffer was proportional to the size of tissue section. The sample was bathed at 60°C for 1 h, and then inactivated at 98°C for 10 min. After cooling, the sample was centrifuged (16,000 × g, 2 min) and the supernatant was transferred to a new tube. One to two microliter of the supernatant was used as the template in a 20 μL PCR reaction. ➂ PCR reaction conditions: pre-denaturation at 95°C for 3 min; denaturation at 94°C for 30 s; annealing at 55–60°C for 25–30 s; extension at 72°C for 30–50 s; 35 cycles followed by repair-extension at 72°C for 5–8 min. ➃ PCR reaction: one to two microliter of template DNA at 20–50 ng/μL; forward primer 10 μM, 2 μL; reverse primer10 μM, 2 μL; dNTP (mix) 10 mM, 2 μL; 10 × Taq Buffer (with MgCl_2_), 5 μL Taq enzyme 5 U/μL, 0.5 μL; Add ddH_2_O to 50 μL.

### Genotyping of DNA Samples

The rs1059057 locus of *SP-A1* gene was genotyped by PCR-SSCP method. The results were compared with the normal sequences of Gen Bank gene pool and analyzed by sequence analysis software.

### Statistical Analysis

The data were analyzed by SPSS 22.0 statistical software. The sex, mode of delivery, and regularity of lung maturation in the two groups were tested by *X*^2^-test. Birth weight and gestational age were tested by *t*-test, and the ratio of gene polymorphism rs1059057 in *SP-A1* was tested by *X*^2^-test. Statistical significance was considered at *P* < 0.05. A power calculation on the G^*^Power program was also performed, based on Cohen's method. When an effect size index of 0.2 (corresponding to “weak to moderate” gene effect) was used the present sample size revealed a >93% power for detection of significant association (*a* < 0.05).

## Results

No significant difference in sex, gestational age, birth weight, mode of birth, cesarean section, and regular lung maturation was observed between the case and the control groups (*P* > 0.05) (Table 1).

**Table 1:** Comparison of general data of the case-control groups.

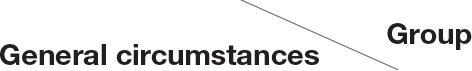	**Number**	**Case**	**Control**	***x^**2**^*/*t***	***P***
Gender (male/female)	120	58/62	56/64	0.067	0.800
Gestational age (x ±*s*,w)	120	30.40 ± 0.70	30.56 ± 0.66	−1.736	0.084
Birth weight (x ±*s*,g)	120	1584.75 ± 139.01	1620.83 ± 161.34	−1.856	0.065
Maternal pregnancy hypertension (yes/no)	120	53/67	42/78	0.000	1.000
Premature rupture of membranes (yes/no)	120	35/85	40/80	0.000	1.000
Intramuscular injection of dexamethasone (yes/no)	120	84/36	79/41	0.478	0.489
Cesarean section (yes/no)	120	64/56	57/63	0.817	0.366

*Statistical significance was considered at P < 0.05*.

Two genotypes of *A/G and A/A* were detected at *SP-A1 rs1059057* locus in both the case and control group. In the case group, the frequencies of the two genotypes were 53 and 47%, and the frequencies of *A* allele and *G* allele were 73 and 27%, respectively. In the control group, the frequencies of the two genotypes were 42 and 58%, and the frequencies of *A* allele and *G* allele were 79 and 21%, respectively. No significant difference was observed in the genotype frequency of *SP-A1* (rs1059057) locus between the case and the control groups (*X*^2^ = 3.275, *P* > 0.05). No significant difference was observed neither in the allele frequencies between the case and the control groups (*X*^2^ = 2.255, *P* > 0.05) (Table 2).

**Table 2:** Distribution of alleles and genotypes of *SP-A1* rs1059057 locus in two groups (cases, %).

**Group**	**Number**	**Genotypic frequency**		**Allele frequency**	
		***AA***	***AG***	***A***	***G***
Case	120	56 (47)	64 (53)	176 (73)	64 (27)
Control	120	70 (58)	50 (42)	190 (79)	50 (21)
*x*^2^		3.275		2.255	
*P*		0.070		0.133	

*Statistical significance was considered at P < 0.05*.

## Discussion

Alveolar surfactant (PS) is a complex composed of lipids and special proteins synthesized and secreted by type II alveolar epithelial cells. It contains four protein components, SP-A, SP-B, SP-C, and SP-D, which play different roles based on functional and structural differences. They can not only reduce alveolar surface tension, but also participate in innate immunity (12). The lack of pulmonary surfactant can lead to the increase of alveolar surface tension and alveolar rupture, and affect the ventilation function of lung tissue, which has been considered to be the main cause of NRDS by previous studies, especially for very early births of younger gestational age (13). SP-A is a protein encoded by the *SFTPA1* gene on the long arm of chromosome 10, which is a member of the C-lectin subfamily (14, 15) and play an important role in regulating the homeostasis of pulmonary surfactants and preventing the invasion of respiratory pathogens by binding lipids and carbohydrates on the surface of microorganisms (16). SP-A is one of the most abundant surface active proteins secreted by type II alveolar epithelial cells, and its deficiency affects the normal function of pulmonary and the metabolism of alveolar surfactant (17). SP-A can participate in the inflammatory response of lung tissue by binding to pathogens (18). Meanwhile SP-A can also reflect the injury of alveolar epithelial cells (19). SP-A1 protein is one of the subtypes of SP-A protein, the deficiency or structural changes of which lead to abnormal alveolar function and affect the normal function of lung tissue. It was found that mice lacking only SP-A did not develop RDS after full-term birth (20), while mice with the *SP-A* gene knocked out were more likely to develop pulmonary infection (21). It can be inferred that RDS in very premature infants may occur under the dual effects of decreased lung function and pulmonary inflammation caused by lack of SP-A.

A large number of studies have found that *SP-A* gene polymorphism is related to the occurrence of RDS in premature infants. For example, Jo et al. found that 1A^0^ variants and homozygous 1A^0^/1A^0^ genotypes of *SP-A2* gene had protective effects on RDS (22). The haplotype (6A^2^/1A^0^) of *SP-A1* may be closely related to the occurrence of RDS in an independent population, but this risk is limited only to very premature infants (23). *SP-A1* haplotype 6A^4^ is a susceptible factor for RDS in late Greek preterm infants (24). In a study on the United States population, it was found that some *SP-A* alleles/haplotypes were susceptible factors of RDS, such as (1A^0^, 6A^2^, 1A^0^/6A^2^), and some *SP-A* alleles/haplotypes were protective factors of RDS, such as (1A^5^, 6A^4^, 1A^5^/6A^4^) (25); However, the results obtained in the population study in South Korea were the opposite (22). A Dutch study of twin fetuses found that their haplotypes were not associated with the occurrence of RDS (26). Chang et al. found that the polymorphism of *SP-A* (+186A/G) gene was closely related to the occurrence of RDS in premature infants (27). In addition, our previous studies found that the genotype and allele frequencies of *SP-A1* (SNP) locus (rs1059047, and rs1136450) were not associated with the occurrence of RDS in Mongolian premature infants, and the haploid 6A^2^ of *SP-A1* allele was the susceptible gene of RDS in Mongolian premature infants, and haploid 6A was the protective gene (28). Thus, it can be seen that the association between *SP-A* gene polymorphism and the occurrence of RDS is affected by race or regional environment.

The main purpose of this study is to investigate the relationship between *SP-A1* rs1059057 locus gene polymorphism and Mongolian very premature infants' RDS. Our results showed that there was no significant association between the frequencies of genotypes and alleles of *SP-A1* rs1059057 locus and the incidence of RDS in Mongolian very preterm infants, which is consistent with the results of Dutch twin study (26) and our previous study on *SP-A1* (SNP) locus (rs1059047, rs1136450) (28). However, many previous studies also showed that *SP-A* gene polymorphism was related to the occurrence of RDS in premature infants (23–25, 27), which is inconsistent with our results. Two points are especially worthy of notice. The first is that the populations are different in those studies. The samples selected in our study are very premature Mongolian infants in the western part of Inner Mongolia, and the experimental results may be influenced by the stratification of the population, regional environment, ethnic groups, lifestyle, and other factors. Another point is that the technical approaches and sample size are different. The samples selected in this study were very premature Mongolian infants in western Inner Mongolia, the sample size was relatively small, and the time span of collecting samples was long; therefore, the genotyping method of PCR-SSCP used in the early stage of our research was used throughout the study for the consistency of experimental methods and conditions, which may also have a certain impact on the experimental results.

In a word, in this study we investigated the relationship between the rs1059057 gene polymorphism of *SP-A1* and the RDS of Mongolian very premature infants in western Inner Mongolia, and found that the gene polymorphism of *SP-A1* (rs1059057) was not related to the incidence of RDS in Mongolian very premature infants in western Inner Mongolia. Of course, it is necessary to increase the sample size and adopt more advanced and more sensitive detection methods to verify our current results in the future.

## Data Availability Statement

All datasets generated for this study are included in the article/supplementary material.

## Ethics Statement

The studies involving human participants were reviewed and approved by the Medical Ethics Committee of the affiliated Hospital of Inner Mongolia Medical University. Written informed consent to participate in this study was provided by the participants' legal guardian/next of kin.

## Author Contributions

HM, CA, CL, YayZ, YanZ, and CX has made contributions to the research, design, revision and finalization of manuscripts. XW and YuZ contributed to the analysis and interpretation of the data and the drafting of the manuscript.

### Conflict of Interest

The authors declare that the research was conducted in the absence of any commercial or financial relationships that could be construed as a potential conflict of interest.
